# A prognostic score for patients with acute-on-chronic liver failure treated with plasma exchange-centered artificial liver support system

**DOI:** 10.1038/s41598-021-81019-8

**Published:** 2021-01-14

**Authors:** Lingyao Du, Yuanji Ma, Shaoqun Zhou, Fang Chen, Yan Xu, Ming Wang, Xuezhong Lei, Ping Feng, Hong Tang, Lang Bai

**Affiliations:** Center of Infectious Diseases, West China Hospital of Sichuan University, No.37 GuoXue Xiang, Wuhou District, Chengdu, 610041 China

**Keywords:** Continuous renal replacement therapy, Risk factors

## Abstract

Artificial liver support system (ALSS) therapy is widely used in patients with hepatitis B virus-related acute-on-chronic liver failure (HBV-ACLF). We aimed to develop a predictive score to identify the subgroups who may benefit from plasma exchange (PE)-centered ALSS therapy. A total of 601 patients were retrospectively enrolled and randomly divided into a derivation cohort of 303 patients and a validation cohort of 298 patients for logistic regression analysis, respectively. Five baseline variables, including liver cirrhosis, total bilirubin, international normalized ratio of prothrombin time, infection and hepatic encephalopathy, were found independently associated with 3-month mortality. A predictive PALS model and the simplified PALS score were developed. The predicative value of PALS score (AUROC = 0.818) to 3-month prognosis was as capable as PALS model (AUROC = 0.839), R score (AUROC = 0.824) and Yue-Meng’ score (AUROC = 0.810) (all *p* > 0.05), and superior to CART model (AUROC = 0.760) and MELD score (AUROC = 0.765) (all *p* < 0.05). The PALS score had significant linear correlation with 3-month mortality (R^2^ = 0.970, *p* = 0.000). PALS score of 0–2 had both sensitivity and negative predictive value of > 90% for 3-month mortality, while PALS score of 6–9 had both specificity and positive predictive value of > 90%. Patients with PALS score of 3–5 who received 3–5 sessions of ALSS therapy had much lower 3-month mortality than those who received 1–2 sessions (32.8% vs. 59.2%, *p* < 0.05). The more severe patients with PALS score of 6–9 could still benefit from ≥ 6 sessions of ALSS therapy compared to ≤ 2 sessions (63.6% vs. 97.0%, *p* < 0.05). The PALS score could predict prognosis reliably and conveniently. It could identify the subgroups who could benefit from PE-centered ALSS therapy, and suggest the reasonable sessions.

**Trial registration:** Chinese Clinical Trial Registry, ChiCTR2000032055. Registered 19th April 2020, http://www.chictr.org.cn/showproj.aspx?proj=52471.

## Introduction

Acute-on-chronic liver failure (ACLF) is a progressive disease associated with rapid clinical deterioration and high mortality. In Asian, hepatitis B virus (HBV) infection accounts for the majority of precipitating factors in ACLF^1^. The accumulation of various toxins and inflammatory cytokines leads to life-threatening complications in patients with ACLF^2,3^. Several large, prospective multicentre studies have shown that patients suffering from ACLF encounter extremely poor prognosis with the 28-day mortality of 30–90%^1,4,5^. Current medicine treatment involves the management of the precipitating events and treatment of complications until the liver eventually recovers, and liver transplantation effectively treats patients with ACLF who respond poorly to the medicine treatment^2,3^. However, liver transplantation is limited by organ scarcity and complicated recipients selection. Over the past three decades, artificial liver support system (ALSS) therapy has been developed to be an therapeutic option as removal of toxins improves the capacity of the liver to regenerate. Some studies found that ALSS therapy could improve the short-term prognosis of patients with ACLF^6–9^, especially under the mode of plasma exchange (PE)^7,8^. Thus, PE has been one of the recommended therapeutic methods for patients with liver failure in China and USA^10–12^. Even if some studies considered it useless^13–16^, it is still a safe, well tolerated, and useful bridge to liver transplantation until a suitable organ is available^17–19^. A possible reason of conflicting results is the selection of patients. Xia et al. found that patients with HBV-related ACLF (HBV-ACLF) and lower Model for End-stage Liver Disease (MELD) scores had significantly better outcomes than those with HBV-ACLF and higher MELD scores^20^. Other studies also suggested that ALSS therapy afforded survival benefits in specific subgroups^21,22^. Thus, subgroups of patients with HBV-ACLF who could benefit from PE-centered ALSS therapy and factors affecting survival must be identified.

To guide and optimize the targeted therapy for patients with HBV-ACLF, a practical, accurate decision-making tool is urgently needed to help clinicians evaluate risks and decide whether to initiate PE-centered ALSS therapy or to prepare for LT as soon as possible. Huang et al. used classification and regression tree (CART) analysis and found that HBV-ACLF patients with a prothrombin time (PT) ≤ 27.8 s but hepatic encephalopathy (HE) may benefit from PE-centered ALSS therapy, especially when the total bilirubin level was ≤ 455 μmol/L^23^. Here, multivariable logistic regression analysis was applied to develop a predictive model and a simplified, easy-to-use, bedside score. We hope the model and score helps clinicians to identify patients at different levels of risks and screen patients eligible for PE-centered ALSS therapy. We also compared the accuracy of our predictive model and score in predicting 3-month mortality with several earlier predictive models, including the CART model^23^, MELD score^24^, R score^22^, and Yue-Meng’ score^25^.

## Materials and methods

### Study design and patients

**Figure 1:**
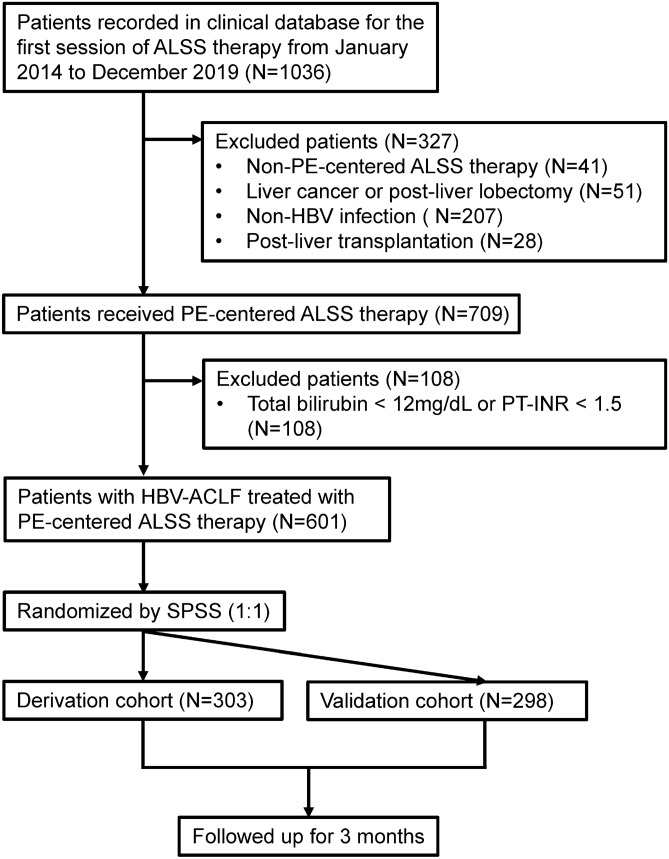
Flow diagram of patient selection. Of the 1036 patients in our database that received ALSS therapy, 435 were excluded from the study. The remaining 601 patients with HBV-ACLF were randomly divided into a derivation cohort (N = 303) and a validation cohort (N = 298). *ALSS* artificial liver support system, *PE* plasma exchange, *HBV* hepatitis B virus, *ACLF* acute-on-chronic liver failure, *SPSS* SPSS v.22 (IBM SPSS).

Patients who received ALSS therapy in the Center of Infectious Diseases, West China Hospital of Sichuan University were consecutively recorded in a clinical database since January 2014. Patients were evaluated on a case-by-case basis by treating physician, and arranged for ALSS therapies only if they had at least one of the following indications: liver failure or pre-liver failure, severe hyperbilirubinemia with no response to medicine, and perioperative period of liver transplantation for end-stage liver disease^11^. Patients between January 2014 and December 2019 were retrospectively included in this study (N = 1036; Fig. 1). Patients treated with non-PE-centered ALSS therapy (N = 41) were excluded. Patients who received liver transplantation before the first session of PE-centered ALSS therapy were excluded (N = 28). Patients with liver cancer or post-liver lobectomy (N = 51), or without HBV infection (N = 207) were also excluded. Patients who did not meet the HBV-ACLF criteria (108) were excluded too. The remaining 601 patients were randomly divided into two cohorts: a derivation cohort (N = 303) and a validation cohort (N = 298). All patients were followed up for 3 months after the first session of PE-centered ALSS therapy. During the follow-up time, if a patient underwent liver transplantation, he/she was considered dead.

Regardless of the presence of cirrhosis, patients with chronic HBV infection, total bilirubin (TBil) ≥ 12 mg/dL and international normalized ratio (INR) of PT (PT-INR) ≥ 1.5 were diagnosed with HBV-ACLF^4^. The severity of HBV-ACLF was rated according to MELD score^24^. The diagnosis of liver cirrhosis (LC) was based on ultrasound and/or computed tomography (CT). Ascites was diagnosed by physical examination, ultrasound and/or CT. HE was defined as neuropsychiatric abnormalities including the cognitive, affective, behavior and consciousness, and the brain edema was identified by CT^26^. Hepatorenal syndrome (HRS) was defined as acute renal failure according to the criteria created by the International Club of Ascites^27^. Spontaneous bacterial peritonitis (SBP) was diagnosed by clinical manifestations, laboratory examination of ascites and procalcitonin.

The study was approved by the Biomedical Research Ethics Committee of West China Hospital of Sichuan University. All research contents were implemented in accordance with the ethical standards stipulated in the Declaration of Helsinki in 1964 and its subsequent amendments. Informed consent was obtained from each participant or his/her parent or legal guardian.

### PE-centered ALSS therapy

All patients received standard medicine treatment and PE-centered ALSS therapy. The standard medication included antiviral drugs, hepatoprotective agents, and drugs to treat complications. The composition of PE-centered ALSS therapy was plasma adsorption therapy for 2 h followed immediately by PE therapy for nearly an hour, with the use of continuous renal replacement therapy (CRRT) if necessary. Plasma adsorption was composed with plasma bilirubin adsorption and plasma perfusion namely double plasma molecular adsorption system (DPMAS) therapy^28^. Fresh frozen plasma and/or ordinary plasma as the replacement fluid with a dose of half the total plasma volume of a patient (approximately 1500 mL) was used in PE therapy. The DPMAS plus PE therapy was performed every 1–2 days. If patients had grade III/IV HE or indications of CRRT, they received DPMAS plus PE therapy along with CRRT. Patients received regional citrate anticoagulation or heparin anticoagulation. ALSS therapy was discontinued due to one of the following conditions: improvement of patient's condition and TBil < 10 mg/dL with reduced PT-INR, conditions that did not allow further ALSS therapy, or the ones who refused to receive further ALSS therapy.

### Statistical analysis

Patients were randomly divided into a derivation cohort and a validation cohort using SPSS software (IBM SPSS) with a ratio of 1:1. The *t* test and the *U* test were performed to calculated differences between quantitative data of normal distribution and that of non-normal distribution, respectively. The chi-squared test or Fisher’s exact test was performed to calculate differences between qualitative data. The predictors for 3-month prognosis in the derivation cohort were analyzed by logistic regression in univariate analysis. For any variables with *p* ≤ 0.1 in the univariate analysis, the backward stepwise (likelihood ratio) method was performed in a multivariate analysis. The predictors obtained from the derivation cohort were tested in the validation cohort. Predictive factors with an area under the receiver operating curves (AUROCs) > 0.750 in the derivation cohort that was equivalent or greater in the validation cohort was used to derive our predictive model. With the purpose of deriving a simple, specific predictive score for patients treated with PE-centered ALSS therapy, we included clinically relevant characteristics and laboratory parameters observed at baseline. An ordinal grading (0–2) was carried out for individual parameters by comprehensively considering their cut-off values of AUROCs predicting the probability of 3-month prognosis, ACLF diagnostic criteria^5,29^, and clinically significant values. A score was obtained by combining the individual grade of all the significant parameters. The score was further used for a grading system by using the proportion of probability for: (1) 3-month mortality, and (2) at least with < 20%, 30–50% and > 80% margins across the grade for the outcome. Multiple comparisons of the score with other predictors were carried out by AUROC. Statistical significance was set at *p* < 0.05. The statistical tests were performed using SPSS v.22 (IBM SPSS), except multiple comparisons of AUROCs, which were performed using MedCacl v.19 (MedCalc Software). The figures of AUROCs and linear regression lines were drawn using MedCacl v.19 (MedCalc Software) and GraphPad Prism 7 (GraphPad Software Inc.), respectively.

## Results

### Patient characteristics

A total of 601 patient were enrolled and randomly divided into a derivation cohort (N = 303) and a validation cohort (N = 298) with a ratio of 1:1 through SPSS software. There were no significant differences between the two cohorts in gender, age, the proportion of LC, causes of liver disease, and laboratory parameters before the first session of PE-centered ALSS therapy (Table 1). The MELD scores were similar in the two cohorts. There was no significant difference in median sessions of PE-centered ALSS therapy. The overall 3-month mortality was not significantly different between the two cohorts.

**Table 1:** Characteristics of derivation and validation cohorts.

	Derivation cohort (N = 303)	Validation cohort (N = 298)	*p*
Female	27 (8.9%)	26 (8.7%)	0.936
Age (years)	43.2 ± 11.1	42.9 ± 10.7	0.759
Liver cirrhosis	232 (76.6%)	230 (77.2%)	0.858
**Etiology**			0.080
HBV infection only	216 (71.3%)	231 (77.5%)	
HBV infection plus other precipitating factors	87 (28.7%)	67 (22.5%)	
**Antiviral agents**			0.419
Entecavir	224 (73.9%)	209 (70.1%)	
Tenofovir	70 (23.1%)	75 (25.2%)	
Others	9 (3.0%)	14 (4.7%)	
**Duration of antiviral therapy**			0.687
≥ 2 weeks	96 (31.7%)	99 (33.2%)	
< 2 weeks	207 (68.3%)	199 (66.8%)	
HBV DNA (log10 IU/mL)	4.25 (3.14–5.84)	4.56 (3.01–5.95)	0.985
HBV DNA ≥ 3 log10 IU/mL	249 (82.2%)	240 (80.5%)	0.605
MELD score	27.2 ± 5.5	26.6 ± 4.97	0.217
**Infection**			0.578
No SBP	87 (28.7%)	89 (29.9%)	
SBP only	76 (25.1%)	64 (21.5%)	
SBP plus other site infection	140 (46.2%)	145 (48.7%)	
Hepatic encephalopathy	66 (21.8%)	69 (23.2%)	0.687
**Grade of hepatic encephalopathy**			0.603
None	237 (78.2%)	230 (77.2%)	
I–II	51 (16.8%)	57 (19.1%)	
III–IV	15 (5.0%)	11 (3.7%)	
PT-INR	2.08 (1.77–2.50)	2.02 (1.74–2.47)	0.171
PT-INR ≥ 2.0	178 (58.7%)	153 (51.3%)	0.068
Serum creatinine (μmol/L)	81 (69–100)	81 (70–97)	0.838
Serum creatinine (× ULN)^a^	0.76 (0.67–0.94)	0.77 (0.67–0.92)	0.783
**Serum creatinine (mg/dL)**			0.122
Male: < 1.2; Female: < 1.0	260 (85.8%)	268 (89.9%)	
Male: ≥ 1.2; Female: ≥ 1.0	43 (14.2%)	30 (10.1%)	
Total bilirubin (μmol/L)	442.5 ± 116.4	443.8 ± 122.5	0.895
Total bilirubin ≥ 425 μmol/L	156 (51.5%)	154 (51.7%)	0.962
Direct bilirubin (μmol/L)	297.6 ± 93.1	305.9 ± 98.9	0.287
Direct bilirubin to total bilirubin ratio	0.72 (0.65–0.79)	0.72 (0.65–0.78)	0.708
Alanine aminotransferase (IU/L)	137 (63–325)	124 (73–248)	0.825
Aspartate aminotransferase (IU/L)	136 (93–251)	135 (91–231)	0.702
Aspartate aminotransferase to alanine aminotransferase ratio	1.11 (0.68–1.83)	1.13 (0.74–1.63)	0.839
Albumin (g/L)	31.8 ± 3.8	32.1 ± 4.0	0.363
Albumin to globulin ratio	1.31 ± 0.46	1.31 ± 0.47	0.894
Serum sodium (mmol/L)	135.2 ± 4.2	135.1 ± 4.4	0.896
Serum potassium (mmol/L)	3.62 ± 0.56	3.67 ± 0.65	0.376
Serum chloride (mmol/L)	96.2 ± 5.2	96.7 ± 4.7	0.457
Venous blood ammonia (mmol/L)	67 (49–94)	68 (50–95)	0.639
Hemoglobin (g/L)	119 ± 20	118 ± 20	0.727
Platelets (× 10^9^/L)	85 (60–114)	84 (61–118)	0.922
White blood cells (× 10^9^/L)	6.8 (5.2––9.4)	6.6 (5.1–9.0)	0.299
Median sessions of ALSS therapy	4 (3–5)	4 (3–5)	0.387
3-month mortality	122 (40.3%)	102 (34.2%)	0.126

*HBV* hepatitis B virus, *ACLF*, acute-on-chronic liver failure, *MELD* model for end-stage liver disease, *SBP* spontaneous bacterial peritonitis, *PT-INR* international normalized ratio (INR) of prothrombin time (PT), *ULN* upper limit of normal, *ALSS* artificial liver support system.

^a^Serum creatinine (× ULN) equals to serum creatinine (μmol/L) divided by the upper limit of normal (Male: 106 μmol/L; Female: 88 μmol/L). Measurement data are represented as mean ± SD (Normally distributed data) or median (P25–P75) (Non-normally distributed data). Enumeration data are represented as frequencies (proportion).

### Development of PALS model in derivation cohort

The derivation cohort was analyzed for the predictors of 3-month prognosis on the basis of baseline parameters by logistic regression analysis. In multivariate analysis, five baseline variables before ALSS therapy, LC, TBil, international normalized ratio (INR) of PT (PT-INR), infection, and HE, were found to be independently associated with 3-month prognosis (Table 2). A predictive PALS model, Logit (*P*) =  − 7.498 + 0.878 × LC + 0.006 × TBil + 1.268 × PT-INR + 0.529 × infection + 1.506 × HE, was developed by using multivariate logistic regression analysis in backward stepwise (Likelihood Ratio) method.

**Table 2:** Predictors for 3-month prognosis in derivation cohort.

	Univariate			Multivariate 1			Multivariate 2		
	HR	95% CI	*p*	HR	95% CI	*p*	HR	95% CI	*p*
Female	1.42	0.64–3.143	0.383						
Age (years)	1.02	1.00–1.05	0.030						
Liver cirrhosis	2.63	1.44–4.80	0.002	2.41	1.10–5.27	0.028	2.65	1.18–5.93	0.018
**Etiology**									
HBV infection only	1	–	–						
HBV infection plus other precipitating factors	0.94	0.57–1.56	0.802						
HBV DNA (log10 IU/mL)	1.02	0.89–1.17	0.803						
HBV DNA ≥ 3 log10 IU/mL	1.30	0.70–2.40	0.402						
Antiviral agents	0.93	0.59–1.45	0.745						
**Duration of antiviral therapy**									
≥ 2 weeks	1	–	–						
< 2 weeks	1.11	0.68–1.82	0.677						
Total bilirubin (μmol/L)	1.01	1.00–1.01	0.000	1.01	1.00–1.01	0.000	1.01	1.00–1.01	0.000
Direct bilirubin (μmol/L)	1.00	1.00–1.01	0.032						
Alanine aminotransferase (IU/L)	1.00	1.00–1.00	0.573						
Aspartate aminotransferase (IU/L)	1.00	1.00–1.00	0.638						
Albumin (g/L)	0.96	0.91–1.03	0.246						
Globulin (g/L)	1.00	0.99–1.01	0.784						
Serum creatinine (× ULN)^a^	3.034.25	2.15–8.42	0.000						
Serum sodium (mmol/L)	0.96	0.91–1.01	0.146						
Serum potassium (mmol/L)	1.13	0.66–1.92	0.652						
Serum chloride (mmol/L)	0.98	0.95–1.02	0.328						
Venous blood ammonia (mmol/L)	1.01	1.00–1.01	0.013						
PT-INR	4.93	2.98–8.15	0.000	3.55	2.04–6.20	0.000	3.86	2.15–6.94	0.000
Hemoglobin (g/L)	0.98	0.97–1.00	0.032						
Platelets (× 10^9^/L)	0.99	0.98–0.99	0.000						
White blood cells (× 10^9^/L)	1.13	1.03–1.23	0.008						
Infection	3.26	1.84–5.79	0.000	1.70	1.18–2.45	0.005	1.87	1.28–2.73	0.001
Hepatic encephalopathy	8.86	4.61–17.02	0.000	4.51	2.18–9.31	0.000	4.13	1.94–8.83	0.000
Sessions of ALSS therapy	0.75	0.64–0.87	0.000				0.72	0.60–0.85	0.000

*HR* hazard ratio, *CI* confidence interval, *HBV* hepatitis B virus, *PT-INR* international normalized ratio (INR) of prothrombin time (PT), *ULN* upper limit of normal, *ALSS* artificial liver support system.

^a^Serum creatinine (× ULN) equals to serum creatinine (μmol/L) divided by the upper limit of normal (Male: 106 μmol/L; Female: 88 μmol/L).

### Testing of PALS model in validation cohort

The five independent predictors were tested in validation cohort before developing a simplified predictive score. LC, TBil, PT-INR, infection and HE were also found to be the independent predictors of 3-month prognosis in multivariate analysis (Table 3). The PALS model had a good predictability with AUROCs of 0.839 (95% CI 0.795–0.883, *p* = 0.000) and 0.800 (95% CI 0.746–0.854, *p* = 0.000) in derivation and validation cohorts, respectively. The expected and observed 3-month mortality from derivation cohort (R^2^ = 0.967, *p* = 0.000) matched with the validation cohort (R^2^ = 0.935, *p* = 0.000; Suppl. Fig. S1A).

**Table 3:** Testing predictors for 3-month prognosis in validation cohort.

	Multivariate 1			Multivariate 2		
	HR	95% CI	*p*	HR	95% CI	*p*
Liver cirrhosis	2.40	1.12–5.15	0.024	2.53	1.17–5.47	0.019
Total bilirubin (μmol/L)	1.01	1.00–1.01	0.000	1.01	1.00–1.01	0.000
PT-INR	2.48	1.42–4.31	0.001	2.37	1.35–4.17	0.003
Infection	1.82	1.27–2.60	0.001	1.86	1.30–2.67	0.001
Hepatic encephalopathy	3.50	1.79–6.87	0.000	3.23	1.63–6.43	0.001
Sessions of ALSS therapy				0.84	0.71–0.98	0.031

*HR* hazard ratio, *CI* Confidence interval, *PT-INR* international normalized ratio (INR) of prothrombin time (PT), *ALSS* artificial liver support system.

### Development of PALS score and PALS grade

With good applicability of the predictive model, the five individual parameters were scored. An ordinal grading (0–2) with distinct hazard ratios on logistic regression was carried out by comprehensively considering their cut-off values of AUROCs predicting the probability of 3-month prognosis, ACLF diagnostic criteria, and clinically significant values (Suppl. Table S1, Table 4). The total PALS score ranges from a minimum of 0 to a maximum of 9. The score was used for a grade system: Grade I for a score of 0–2, Grade II for 3–5 and Grade III for 6–9 with the 3-month mortality of 5.7%, 44.8% and 84.3% in derivation cohort, respectively. The scoring was for easy-to-recollect laboratory parameters or the clinical features with a distinct hazard ratio on logistic regression in derivation and validation cohorts (all hazard ratio > 2 and all *p* = 0.000; Suppl. Table S2).

**Table 4:** PALS score and PALS grade.

PALS score					
Points	Liver cirrhosis	Total bilirubin (μmol/L)	PT-INR	Infection	Hepatic encephalopathy
0	No	< 425	< 2.0	No SBP	No
1	Yes	425–650	2.0–2.5	SBP only	I–II
2	–	≥ 650	≥ 2.5	SBP plus other site infection	III–IV
Minimum 0, maximum 9					

PALS grade					
Grade					Score
I					0–2
II					3–5
III					6–9

*PALS score* predictive score of short-term prognosis for patients treated with artificial liver support system therapy, *PALS grade* grade of PLAS score, *PT-INR* international normalized ratio (INR) of prothrombin time (PT), *SBP* spontaneous bacterial peritonitis.

**Figure 2:**
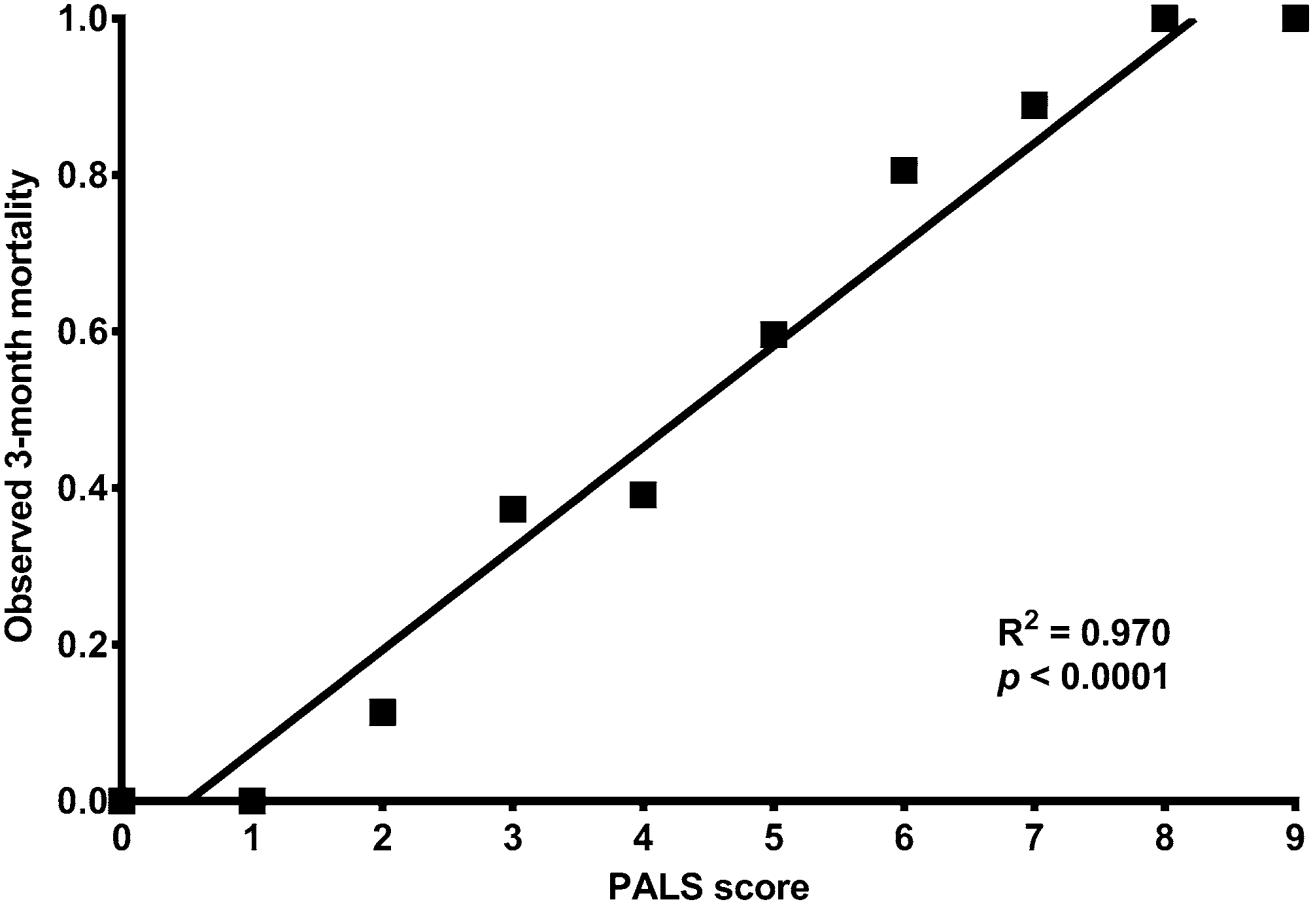
Linear regression lines of PALS score and observed 3-month mortality in the derivation cohort. A linear regression equation was developed for the PALS score and 3-month mortality in derivation: 3-month mortality = 13.0% × PALS score − 6.6%. *PALS score* predictive score of short-term prognosis for patients treated with artificial liver support system therapy.

The PALS scores and 3-month mortality showed an obvious linear correlation in derivation cohort (R^2^ = 0.970, *p* = 0.000; Fig. 2). A linear regression equation was established: 3-month mortality = 13.0% × PALS score − 6.6%. The expected and observed 3-month mortality based on the PALS scores in the derivation cohort (R^2^ = 0.870, *p* = 0.000) matched those of the validation cohort (R^2^ = 0.848, *p* = 0.000; Suppl. Fig. S1B).

### Evaluation of PALS model and PALS score as predictors of 3-month prognosis in derivation and validation cohorts

**Figure 3:**
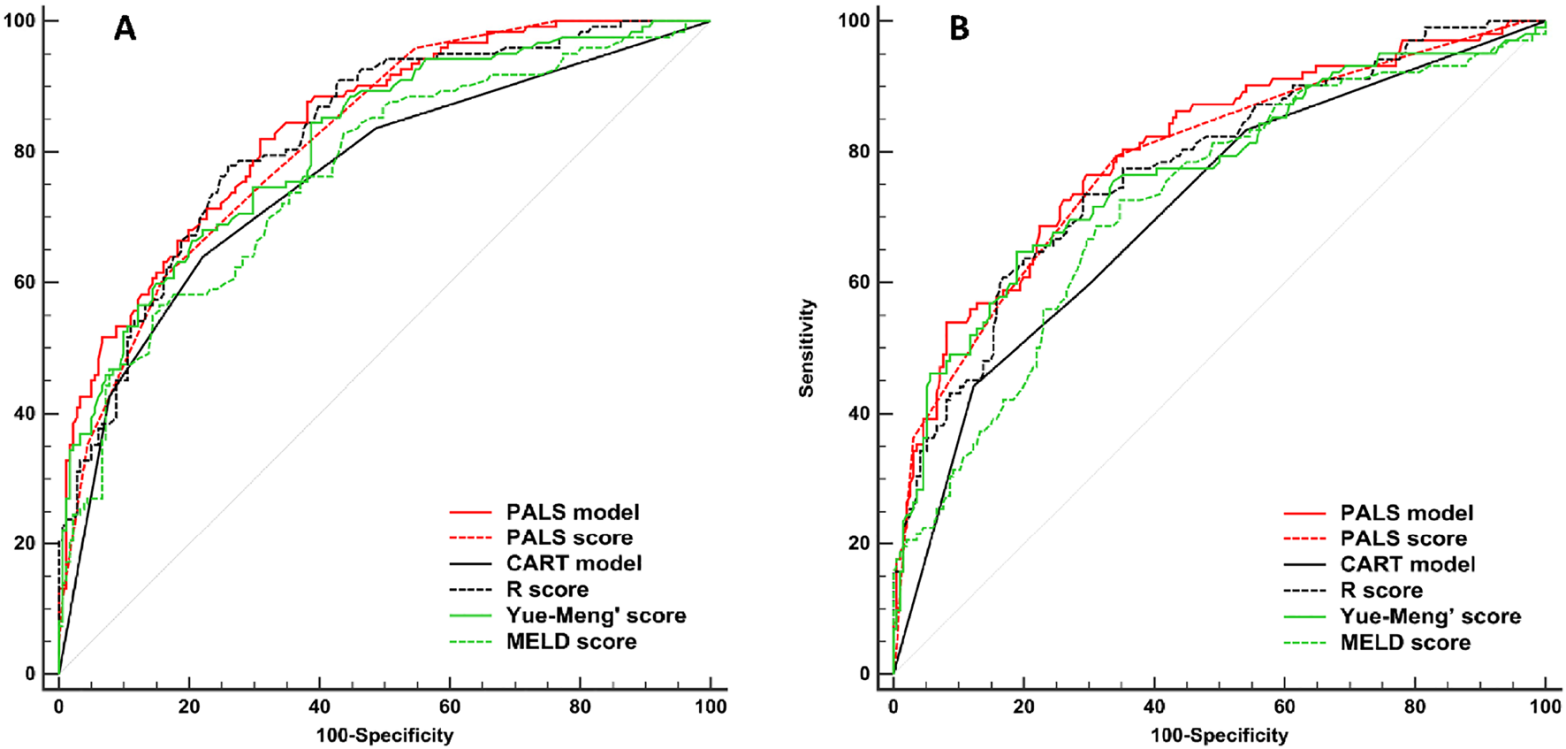
Receiver operating curves (ROC) for the abilities of risk models to predict 3-month mortality. ROC for risk models predicting 3-month mortality in the derivation cohort (**A**) and validation cohort (**B**). Our PALS model and PALS score were as capable or superior to all other models in predicting 3-month mortality. *PALS model* predictive model of short-term prognosis for patients treated with artificial liver support system therapy, *PALS score* predictive score of short-term prognosis for patients treated with artificial liver support system therapy, *CART model* classification and regression tree model, *MELD score* score of model for end-stage liver disease.

PALS model and its simplified PALS score were compared with other predictors, such as CART model, R score, Yue-Meng’ score, and MELD score (Fig. 3, Table 5). AUROCs of PALS model and PALS score in derivation cohort were 0.839 and 0.818, and that in validation cohort were 0.800 and 0.786, respectively. PALS model and PALS score were found to be superior to CART model (AUROC = 0.760), and MELD score (AUROC = 0.765) in predicting 3-month prognosis (all *p* < 0.05). PALS model and PALS score were found to be as capable as R score (AUROC = 0.824), and Yue-Meng’ score (AUROC = 0.810) in predicting 3-month prognosis (all *p* > 0.05). PALS score was found to be as capable as PALS model in predicting 3-month prognosis *p* = 0.093).

**Table 5:** Comparison of the predictive values of PALS model, PALS score and other predictors.

	Derivation cohort				Validation cohort			
	AUROC	95% CI	*Z*	*p*	AUROC	95% CI	*Z*	*p*
PALS model	0.839	0.792–0.878			0.800	0.750–0.844		
PALS score	0.818	0.770–0.860	1.68	0.093	0.786	0.735–0.831	1.49	0.136
CART model	0.760	0.708–0.807	4.72	0.000	0.712	0.657–0.763	4.42	0.000
R score	0.824	0.776–0.865	0.69	0.492	0.775	0.724–0.821	0.91	0.365
Yue-Meng’ score	0.810	0.761–0.853	1.39	0.164	0.769	0.716–0.815	1.26	0.206
MELD score	0.765	0.713–0.812	3.17	0.002	0.721	0.666–0.771	2.83	0.005
PALS score	0.818	0.770–0.860			0.786	0.735–0.831		
CART model	0.760	0.708–0.807	2.61	0.009	0.712	0.657–0.763	3.37	0.001
R score	0.824	0.776–0.865	0.25	0.800	0.775	0.724–0.821	0.39	0.695
Yue-Meng’ score	0.810	0.761–0.853	0.32	0.753	0.769	0.716–0.815	0.67	0.504
MELD score	0.765	0.713–0.812	1.97	0.049	0.721	0.666–0.771	2.24	0.025

Area under the ROC curves (AUROCs) for different models were calculated and compared using the Z test (Delong’s method).

*PALS model* logistic regression model of risk predictors for citrate accumulation, *PALS score* risk score for citrate accumulation, *CI* Confidence interval, *CART model* classification and regression tree model, *MELD*, model for end-stage liver disease.

Most patients with HBV-ACLF who had PALS score of 0–2 or PALS grade I would be survivors with both sensitivity and negative predictive value of > 90%, while most patients with HBV-ACLF who had PALS score of 6–9 or PALS grade III would be victims with both specificity and positive predictive value of > 90% (Table 6).

**Table 6:** Predictive values of PALS score and PALS grade.

	Cut-off	Sensitivity (%)	Specificity (%)	Positive predictive value (%)	Negative predictive value (%)
PALS score	0	100.0	7.2	42.1	100.0
	1	100.0	23.8	46.9	100.0
	2	95.9	45.3	54.2	94.3
	3	75.4	68.5	61.7	80.5
	4	60.7	84.0	71.8	76.0
	5	35.3	95.6	84.3	68.7
	6	11.5	99.5	93.3	62.5
	7	4.9	100.0	100.0	60.9
	8	0.8	100.0	100.0	59.9
	9	0.0	100.0	–	59.7
PALS grade	I	95.9	45.3	54.2	94.3
	II	35.25	95.6	84.3	68.7
	III	0.0	100.0	–	59.7

*PALS score*, predictive score of short-term prognosis for patients treated with artificial liver support system therapy, *PALS grade* grade of PLAS score.

### Correlation between PALS model, PALS score and disease severity

**Figure 4:**
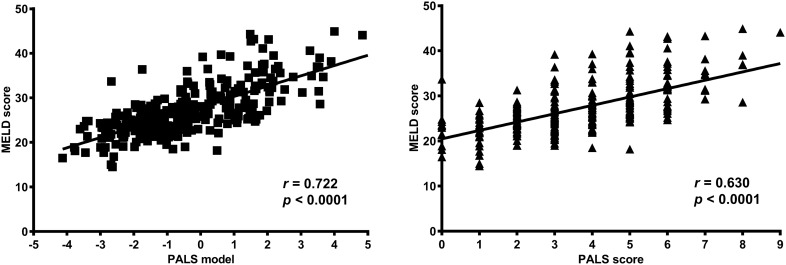
Correlation between PALS model, PALS score and disease severity in derivation cohort. PALS model and PALS score are positively correlated with disease severity rated by MELD score in derivation cohort. *MELD* model for end-stage liver disease, *PALS model* predictive model of short-term prognosis for patients treated with artificial liver support system therapy, *PALS score* predictive score of short-term prognosis for patients treated with artificial liver support system therapy.

PALS model, PALS score were positively correlated with disease severity rated by MELD score in derivation and validation cohorts with all the *p* = 0.000 (Fig. 4).

### Effect of sessions of ALSS therapy on 3-month prognosis in patients with HBV-ACLF

In this study, patients received a median of 4 (3–5) sessions of ALSS therapy (Table 1). In multivariate analysis, sessions of ALSS therapy was also found to be independently associated with 3-month prognosis both in derivation (HR 0.72; 95% CI 0.60–0.85; p = 0.000; Table 2) and validation cohorts (HR 0.84; 95% CI 0.71–0.98; *p* = 0.031; Table 3). For all patients, the ones who received 3–5 sessions of ALSS therapy had much lower 3-month mortality than those who received 1–2 sessions (32.8% vs. 59.2%, *p* < 0.05) (Table 7). However, no significant difference was found although more sessions of ALSS therapy seemed to led to lower mortality (27.7% vs. 32.8%, *p* > 0.05). Then patients were grouped for further analysis. In patients with PALS grade I, the ones who received at least 2 sessions of ALSS therapy had lower 3-month mortality with no significant difference compared to those who received only one session (≤ 16.7% vs. 28.6%, *p* > 0.05). In Patients with PALS grade II, the ones who received 3–5 sessions of ALSS therapy had much lower 3-month mortality than those who received 1–2 session of ALSS therapy (36.6% vs. 58.9%, *p* < 0.05). In patients with PALS grade III, the ones who received ≥ 6 sessions of ALSS therapy had much lower 3-month mortality than those who received 1–2 sessions (63.6% vs. 97.0%, *p* < 0.05), even though these patients still had high mortality.

**Table 7:** Effect of sessions of ALSS therapy on prognosis.

Sessions of ALSS therapy	3-month mortality			
	PALS grade I (n = 175)	PALS grade II (n = 332)	PALS grade III (n = 94)	Total (n = 601)
1	28.6%^a^	84.2%^a^	100.0%^a^	79.5%^a^
2	16.7%^a^	45.9%^a,b^	95.0%^a^	49.4%^b^
3	5.8%^a^	40.8%^b^	90.5%^a^	35.4%^b,c^
4	9.6%^a^	41.9%^b^	77.8%^a^	35.6%^b,c^
5	0.0%^a^	20.4%^b^	72.7%^a^	22.5%^c^
≥ 6	10.0%^a^	27.0%^b^	63.6%^a^	27.7%^c^
1–2	19.4%^a^	58.9%^a^	97.0%^a^	59.2%^a^
3–5	6.5%^a^	36.6%^b^	82.0%^a,b^	32.8%^b^
≥ 6	10.0%^a^	27.0%^b^	63.6%^b^	27.7%^b^

*ALSS* artificial liver support system, *PALS grade* grade of PLAS score, *PALS score* predictive score of short-term prognosis for patients treated with artificial liver support system therapy.

Each superscript letter denotes a subset of PALS Grade categories whose column proportions do not differ significantly from each other at the 0.05 level.

In this study, ALSS therapy was discontinued in 49.3% (297/601) of patients for their condition improved and the ALSS therapy was not further needed. 41.4% of (249/601) patients stopped for refusal of further ALSS therapy, and 9.2% (55/601) of patients stopped because of deteriorated condition under which the patients could not tolerate the treatment. Patients whose condition improved had much lower 3-month mortality than those whose condition not improved (4.4% vs. 69.4%, *p* = 0.000). Among patients without improvement, although the ones who received ≥ 6 sessions of ALSS therapy had much lower 3-month mortality than those who received 1–2 or 3–5 sessions (45.3% vs. 87.5%, *p* < 0.05, or 45.3% vs. 68.4%, *p* < 0.05, respectively), these patients would still encounter high mortality (Suppl. Table S3).

### Ethics approval and consent to participate

This study was approved by the Biomedical Research Ethics Committee of West China Hospital of Sichuan University. Patients were recorded in a previously established clinical database which was approved by the Biomedical Research Ethics Committee of West China Hospital of Sichuan University. Written informed consent was obtained.

## Discussion

In present study, we found that five variables, namely LC, TBil, PT-INR, infection and HE, were the independent predictors of 3-month prognosis in patients with HBV-ACLF treated with PE-centered ALSS therapy. The predictive PALS model and its simplified PALS score had been brought forth. The PALS score could predict 3-month prognosis in these patients reliably and conveniently. Moreover, it could identify the subgroups who could benefit from PE-centered ALSS therapy, and suggest a reasonable session number.

The establishment of our PALS model is based on some classical parameters determining the MELD score^24^, R score^22^, Yue-Meng’ score^25^, CART model^23^, AARC score^29^, CLIF-C ACLF score^5,30^, and HBV-ACLF criteria^4^. Recent studies indicate that the presence of complications is a major risk factor for mortality in patients with HBV-ACLF^31–35^. These findings help to explain the positive correlations between 3-month prognosis and TBil, PT-INR, infection, HE, as well as PALS model and its simplified PALS score. Although contradictory results between the presence of LC and the progress or prognosis of ACLF have been reported previously^36–38^, our study do find that the presence of LC is an independent prognostic risk factor for patients with ACLF along with other studies^39–41^. However, the possible negative correlation between age and prognosis was not found in our study even though age was reported to be an independent risk factor in predicting development of ACLF^36,38^, and decreased capacity of liver regeneration and impaired immune function were reported in older patients^42,43^.

The calculation of PALS model was complicated. For more convenient application, we developed the PALS score, a simplified version which only required common variables and could be used easily at bedside. The predictive value of PALS score was found to be equal to the PALS model, which has been proved as capable as R score and Yue-Meng’ score, and superior to CART model and MELD score in predicting prognosis in our study. Therefore, it has a distinct advantage over these models and scores mentioned above. The simplified scoring system assigns 0 to 2 points for the five parameters, much like the Child-Turcotte-Pugh (CTP) score for patients with LC and the AARC score and CLIF-C ACLF score for patients with ACLF^5,29,30,44^. PALS score is superior to the CART model which is also easy-to-use in predicting 3-month prognosis^23^. The AARC score and CLIF-C ACLF score, two of the most important and easy-to-use prognostic scores for disease severity in patients with ACLF^5,29,30^, probably also have good capability in predicting prognosis in patients with HBV-ACLF treated with PE-centered ALSS therapy because of some of the same parameters (TB, PT-INR, and HE). We had tried to compare the AARC score and CLIF-C ACLF score to our score, but the respiratory indicator (arterial blood gas analysis result) or examination of lactate was missing in many cases in this retrospectively study. The role of AARC score and CLIF-C ACLF score in predicting prognosis in patients with HBV-ACLF treated with PE-centered ALSS therapy requires further investigation.

The short-term mortality rate of HBV-ACLF is extremely high^4^. It is essential to stratify patients by their current condition and possible prognosis to select appropriate treatment strategies: to provide PE-centered ALSS therapy to get recovery, or as a bridge to liver transplantation. The CART model helps physicians identify patients at lower risk (facilitating appropriate application of ALSS as part of a comprehensive treatment), and prioritise liver transplantation for patients at higher risk^23^. The MELD score^24^, R score, AARC score^29^, and CLIF-C ACLF score^5,30^ also shows the capacity to guide such decision-making. In our study, we found that most patients with HBV-ACLF who had PALS score of 0–2 or PALS grade I could benefit with ease from PE-centered ALSS therapy to be survivors, and some patients with PALS score of 3–5 or PALS grade II could also get benefit from more sessions of ALSS therapy. However, patients with PALS score of 6–9 or PALS grade III would still remain poor prognosis due to their serious illness even though they could get benefit from much more sessions of ALSS therapy. It was the same as high mortality among patients with higher ACLF grade on the liver transplant waitlist^45^. Therefore, PE-centered ALSS therapy should only be considered as a bridge to liver transplantation among patients with high PALS score, especially for patients whose condition have not improved after receiving ≥ 6 sessions of ALSS therapy. Liver transplantation should be scheduled in time and performed within 30 days of placement on the waitlist to get a higher than 90% of graft survival probability at 5 years^46^. Some studies have shown that dynamic assessment of prognostic scores could predict outcome better^29,47–49^. It implied that dynamic evaluation of PALS score may be also helpful for real-time clinical decision-making during PE-centered ALSS therapy, but it should be investigated.

Our study has several limitations. First, the PALS score was established in a single center, and the demographic characteristics of patients might not be representative for the general population. Second, the enrolled study subjects were all patients with chronic HBV infection. Whether our results are applicable in patients suffering from ACLF with other precipitating factors remains unclarified. Third, as a retrospective study with a limited data samples, potential confounders may cover the significance of included risk factors. Multicenter, prospective studies with larger study populations are needed to further verify the applicability of PALS score. Lastly, the methodological limitation might also lead to biased results. Cox regression should be considered the preferred analysis method for survival analysis or time-to-event analysis, and logistic regression should be only used as an alternative analysis method. However, the survival status of many early cases of this study only emerged at the end of follow-up. To avoid recalling the sad experience again for the family members, we could not make follow-up calls repeatedly to figure out the survival days of each patient.

In conclusion, PALS score is a validated, user-friendly bedside tool that could reliably screen patients with HBV-ACLF in terms of eligibility for PE-centered ALSS therapy. Most patients with PALS score of 0–2 and some patients with PALS score of 3–5 could benefit from PE-centered ALSS therapy, while patients with PALS score of 6–9 remain high 3-month mortality and liver transplantation should be scheduled in time. Prospective cohort studies are still required to confirm present results.

## Supplementary Information

Supplementary Information.

## Data Availability

The data sets used and/or analyzed during the current study are available from the corresponding author on reasonable request.
