# Supplementary material for: A prognostic score for patients with acute-on-chronic liver failure treated with plasma exchange-centered artificial liver support system
Source: Sci Rep. 2021 Jan 14;11:1469. doi: 10.1038/s41598-021-81019-8 (PMC7809456; doi:10.1038/s41598-021-81019-8)
Supplement: Supplementary file 1 — Supplementary Information. [file 41598_2021_81019_MOESM1_ESM.doc]

**A prognostic score for patients with acute-on-chronic liver failure treated with plasma exchange-centered artificial liver support system**

Lingyao Du^1^, Yuanji Ma^1^, Shaoqun Zhou, Fang Chen, Yan Xu, Ming Wang, Xuezhong Lei, Ping Feng, Hong Tang, Lang Bai

Center of Infectious Diseases, West China Hospital of Sichuan University, Chengdu 610041, China.

^1^These two authors contributed equally to this work.

Corresponding Author: Lang Bai, MD

Center of Infectious Diseases, West China Hospital of Sichuan University

No.37 GuoXue Xiang, Wuhou District, Chengdu 610041, China

Tel: 86-28-85422650

Fax: 86-28-85423052

Email: pangbailang@163.com

Co-Corresponding Author: Hong Tang, MD

Center of Infectious Diseases, West China Hospital of Sichuan University

No.37 GuoXue Xiang, Wuhou District, Chengdu 610041, China

Tel: 86-28-85422650

Fax: 86-28-85423052

Email: htang6198@hotmail.com


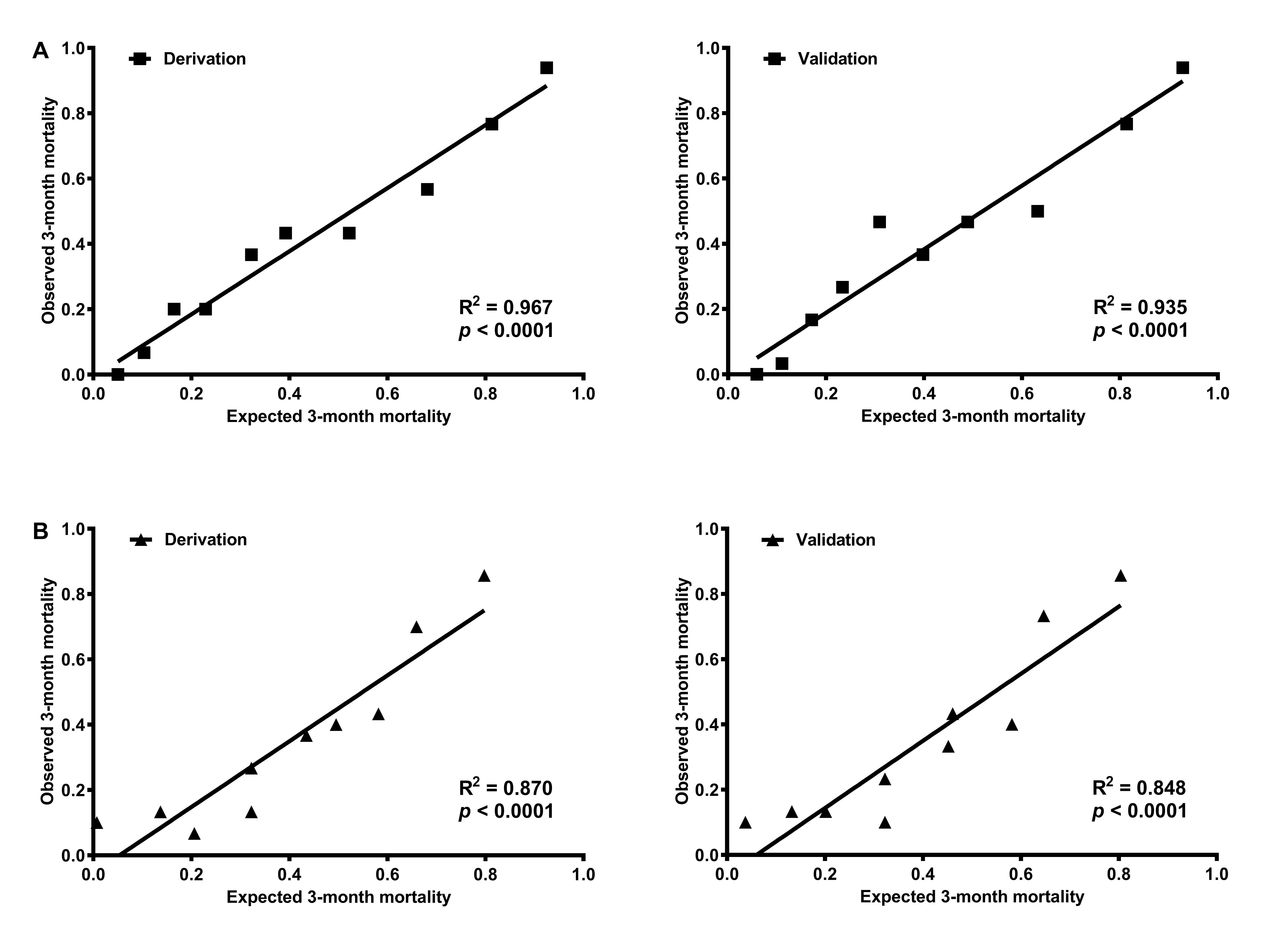


**Suppl. Fig.1 Linear correlation lines of expected 3-month mortality and observed 3-month mortality of PALS model and PALS score.**

(A) The linear correlation lines of expected and observed 3-month mortality in the derivation and validation cohorts based on the PALS model. (B) The linear correlation lines of expected and observed 3-month mortality in the derivation and validation cohorts based on the PALS score. The expected and observed 3-month mortality of the derivation cohort match those of the validation cohort.

Abbreviations: PALS model, predictive model of short-term prognosis for patients treated with plasma exchange-centered artificial liver support system therapy; PALS score, predictive score of short-term prognosis for patients treated with plasma exchange-centered artificial liver support system therapy.

**Suppl. Table 1. Simplified univariate predictors for 3-month prognosis in** **derivation and validation cohort**

|  | Derivation cohort | | | | | |  | | | Validation cohort | | | | |
| --- | --- | --- | --- | --- | --- | --- | --- | --- | --- | --- | --- | --- | --- | --- |
|  | HR | | | 95% CI | *p* | |  |  |  | HR | | 95% CI | *p* | |
| Liver cirrhosis | |  |  | | |  | |  |  | |  | | |  |
| No | | 1 | - | | | - | |  | 1 | | - | | | - |
| Yes | | 2.63 | 1.44～4.80 | | | 0.002 | |  | 1.94 | | 1.04～3.61 | | | 0.036 |
| Total bilirubin (μmol/L) | |  |  | | |  | |  |  | |  | | |  |
| 200～425 | | 1 | - | | | - | |  | 1 | | - | | | - |
| 425～650 | | 2.71 | 1.66～4.44 | | | 0.000 | |  | 2.20 | | 1.31～3.67 | | | 0.001 |
| ≥650 | | 6.42 | 2.13～19.38 | | | 0.001 | |  | 4.15 | | 1.62～10.68 | | | 0.003 |
| PT-INR | |  |  | | |  | |  |  | |  | | |  |
| 1.5～2.0 | | 1 | - | | | - | |  | 1 | | - | | | - |
| 2.0～2.5 | | 2.24 | 1.26～3.97 | | | 0.006 | |  | 2.17 | | 1.20～3.90 | | | 0.010 |
| ≥2.5 | | 7.46 | 3.94～14.13 | | | 0.000 | |  | 4.12 | | 2.23～7.62 | | | 0.000 |
| Infection | |  |  | | |  | |  |  | |  | | |  |
| No SBP | | 1 | - | | | - | |  | 1 | | - | | | - |
| SBP only | | 1.65 | 0.82～3.33 | | | 0.161 | |  | 2.66 | | 1.21～5.86 | | | 0.015 |
| SBP plus other site infection | | 4.64 | 2.52～8.52 | | | 0.000 | |  | 5.31 | | 2.71～10.40 | | | 0.000 |
| Hepatic encephalopathy | |  |  | | |  | |  |  | |  | | |  |
| None | | 1 | - | | | - | |  | 1 | | - | | | - |
| Ⅰ～II | | 6.97 | 3.50～13.89 | | | 0.000 | |  | 4.38 | | 2.39～8.05 | | | 0.000 |
| Ⅲ～Ⅳ | | 33.40 | 4.31～258.89 | | | 0.001 | |  | 29.66 | | 3.72～236.68 | | | 0.001 |

Abbreviations: HR, Hazard ratio; CI, Confidence interval; PT-INR: international normalized ratio (INR) of prothrombin time (PT); SBP, spontaneous bacterial peritonitis.

**Suppl. Table 2. Predictive model for 3-month prognosis in derivation and validation cohorts.**

| Subject | PALS model | | |  | PALS score | | |  | PALS grade | | |
| --- | --- | --- | --- | --- | --- | --- | --- | --- | --- | --- | --- |
|  | HR | 95% CI | *p* |  | HR | 95% CI | *p* |  | HR | 95% CI | *p* |
| Derivation cohort | 2.68 | 2.14～3.39 | 0.000 |  | 2.26 | 1.86～2.75 | 0.000 |  | 9.21 | 5.19～16.34 | 0.000 |
| Validation cohort | 2.31 | 1.87～2.87 | 0.000 |  | 2.03 | 1.68～2.45 | 0.000 |  | 6.15 | 3.66～10.34 | 0.000 |
| Patients with liver cirrhosis | 2.42 | 2.02～2.90 | 0.000 |  | 2.08 | 1.78～2.42 | 0.000 |  | 5.89 | 3.89～8.93 | 0.000 |
| Patients without liver cirrhosis | 2.69 | 1.89～3.83 | 0.000 |  | 2.46 | 1.78～3.39 | 0.000 |  | 24.84 | 7.34～84.09 | 0.000 |
| Patients with HBV infection alone | 2.73 | 1.96～3.80 | 0.000 |  | 2.17 | 1.66～2.84 | 0.000 |  | 8.49 | 3.84～18.78 | 0.000 |
| Patients with HBV infection plus other precipitating factors | 2.43 | 2.03～2.91 | 0.000 |  | 2.14 | 1.83～2.50 | 0.000 |  | 7.19 | 4.64～11.15 | 0.000 |

Abbreviations: PALS model, predictive model of short-term prognosis for patients treated with artificial liver support system therapy; PALS score, predictive score of short-term prognosis for patients treated with artificial liver support system therapy; PALS grade, grade of PALS score; HR, Hazard ratio; CI, Confidence interval.

**Suppl. Table 3. Effect of sessions of ALSS therapy on prognosis of patients whose condition improved or not**

| Sessions of ALSS therapy | 3-month mortality | |
| --- | --- | --- |
|  | Condition improved (n=297) | Condition not improved (n=304) |
| 1 | 0.0% ^a^ | 100.0% ^a^ |
| 2 | 3.1% ^a^ | 79.6% ^a, b^ |
| 3 | 3.7% ^a^ | 77.4% ^a, b^ |
| 4 | 6.7% ^a^ | 71.2% ^b, c^ |
| 5 | 2.3% ^a^ | 47.2% ^c, d^ |
| ≥6 | 4.9% ^a^ | 45.3% ^d^ |
| 1～2 | 2.5% ^a^ | 87.5% ^a^ |
| 3～5 | 4.6% ^a^ | 68.4% ^b^ |
| ≥6 | 4.9% ^a^ | 45.3% ^c^ |

Each superscript letter denotes a subset of patients categories whose column proportions do not differ significantly from each other at the 0.05 level. Abbreviations: ALSS, artificial liver support system therapy.
